# Long-term follow-up and operative performance metrics of inguinal hernioplasty training in the Dominican Republic

**DOI:** 10.1007/s10029-025-03286-y

**Published:** 2025-02-18

**Authors:** Quilvio Colon Diaz, Jose Morel, Cesar Castillo, Alvaro Torres Velasquez, Taty Gisselle Medina Novas, Ryan W. Walters, Corey Lawson, Sardis Sosa, Cynthia Nunez, Jorge Rodriguez, Giampiero Campanelli, David Chen, Charles J. Filipi

**Affiliations:** 1 Hospital L. Bogaert, Mao, Dominican Republic; 2 Hospital Maternidad, Moncion, Dominican Republic; 3 Hospital Regional Universitarion Jose Mana Cabral y Baes, Santiago, Dominican Republic; 4 Institute of Latin American Concern, Santiago, Dominican Republic; 5 Creighton University Department of Clinical Research and Public Health, Omaha, Nebraska USA; 6 Hernia Repair for the Underserved and Chronic Care International, Omaha, Nebraska USA; 7 Hospital Municipal Partido, Dajabon, Dominican Republic; 8 Istituto Clinico Sant’Ambrogio, Milan, Italy; 9 Lichtenstein Amid Hernia Clinic at UCLA, Los Ángeles, California USA; 10 Department of Surgery, CHI Health Creighton University Medical Center, Bergan Mercy Education Building 7710 Mercy Road, Suite 501, Omaha, NE 68124-2368 USA

**Keywords:** Inguinal hernia, Developing country, Long term follow-up

## Abstract

**Purpose:**

The long-term outcomes of inguinal hernioplasty are crucial for evaluating patient benefits, though follow-up can be challenging, especially in low and middle-income countries (LMICs).

**Methods:**

Program coordinators in the Dominican Republic reached out to 288 patients operated on between 2014 and 2019 under a Hernia Repair for the Underserved (HRFU) training initiative. Each patient underwent an anterior Lichtenstein procedure performed by either an international HRFU expert surgeon (128 patients) or two local Dominican surgeons (160 patients) certified using the Operative Performance Rating Scale (OPRS).

**Results:**

Long-term outcomes were obtained from 30% (86/288) of patients. Follow-up data were obtained from 12% (35/288) of patients by a history and physical examination by independent Dominican surgeons. 18% (51/288) completed telephone follow-up using a four-question survey tailored for standard inguinal hernia outcomes. The phone questionnaire follow-up method (18%) was more effective than the H & *P*. One patient required reoperation for a mesh granuloma and one had a reoperation for a recurrent hernia. The average length of follow-up was 52 months.

**Conclusion:**

This study reports the longest durations of follow-up after inguinal hernia repair performed in a LMIC and the longest clinical outcomes follow-up of operations performed using the OPRS training method.

## Introduction

Inguinal hernia affects millions of people worldwide. There are several acceptable techniques for inguinal hernia repair but the modified Lichtenstein tension-free mesh repair, as popularized by Amid [1, 2] remains the surgical treatment of choice in many countries. Hernia recurrence rates have improved with the Lichtenstein mesh repair but chronic postoperative pain remains an issue [3, 4].

Hernia Repair for the Underserved (HRFU) is a non-profit organization focused on delivering high-quality hernia surgery to underserved patients and capacity building in low and middle income countries (LMIC) utilizing structured training and local partnerships. Each mission is designed to simultaneously train local in-country surgeons under a “train the trainer” paradigm, while providing excellent patient care. The surgeon trainee performs 6 sequential surgeries, the first as an assistant to the trainer and the next 5 operations using the operative performance rating scale (OPRS) [5], an American Board of Surgery approved training metric. The OPRS is case specific and includes both metrics related to global surgical skills and detailed queries specific to the steps of the Lichtenstein repair [6, 7]. However, according to personal communication with Dr. Gary Dunnington, one of the OPRS developers, no long term follow-up of outcomes from these OPRS-measured training operations has been utilized to determine OPRS efficacy.

Long-term follow-up remains a challenge even in high income countries. Because of its inherent difficulty, the acquisition of long-term outcomes data has been neglected in LMICs despite good evidence that it is an integral measure of hernioplasty success [8]. Long term follow-up, despite numerous challenges, was obtained on patients operated on by HRFU international expert surgeons and HRFU trained Dominican Republic (DR) surgeons. We describe the successes and challenges of this effort to obtain training and operative outcomes in a LMIC setting. This highlights the importance of pursuing outcomes data no matter the difficulty as an integral part of establishing a system of high-quality surgical care.

## Methodology

In 2014, a HRFU expert trainer, trained two Dominican surgeons selected for the formal HRFU training program. Both surgeons had strong general surgery practices, operated independently, were board certified in their country, and were performing tissue-based inguinal hernia repairs in their daily practice. The trainee surgeons were tracked and accredited based on OPRS metrics and directed feedback on sequential proctored operative cases [5].

During the training program, the expert trainer demonstrated the steps of a modified Lichtenstein repair for each trainee as first assistant on the first training case. Each trainee then performed Lichtenstein repairs with the trainer’s assistance. Using the OPRS, the surgeon trainer provided immediate written and verbal feedback to the trainees regarding areas of proficiency and opportunities for improvement. Surgeons were granted certificates of completion if they achieved an overall OPRS score of 3 or better on each procedure-specific metric by the end of training. The OPRS scoring sheet was provided in both English and Spanish and the trainer was multilingual.

This study followed inguinal hernia patients operated between 2014 and 2019 at two sites in the DR; (1) Mao and Moncion (MM), two adjacent rural towns and (2) the Institute of Latin Concerns (ILAC) in Santiago. Patients were operated upon utilizing the Lichtenstein repair [6, 7]. At MM 160 operations were performed. At ILAC, 128 repairs were performed. The study was designed to obtain long-term follow-up of this cohort of 288 patients. The study is divided into phase I and II at both sites because patient follow-up recruitment was insufficient in phase I but substantially improved with a revised patient recruitment method (phase II).

In 2022, an HRFU Lichtenstein hernia repair follow-up study protocol was designed and study approval was obtained from the Regional Health Service Cibao Occidental, DR and the University of California, Los Angeles Institutional Review Board. An in-country coordinator contacted patients who had undergone a Lichtenstein inguinal hernia repair by the two DR trained surgeons between 2014 and 2019 as part of our training program at Mao and Moncion. One hundred and sixty patients were called. 19% of the listed phone numbers were incorrect and voice mails were left for 7.5% of patients. If the patient did not answer and voicemail was not available with the initial attempted contact, the patient was called two more times and then placed on the uncontacted list (73.5%). The surgeon obtained operative patient informed consent at MM and at ILAC the consent was obtained by an examining physician in the pre-operative clinic. 

### Phase I: In-person history and physical follow-up at Mao & Moncion (MM)

An independent DR board certified surgeon obtained informed consent and then interviewed and examined 14 patients using an HRFU standardized questionnaire and examination form. Electronic spreadsheets were utilized for recording data. The surgeon-examiner and patients were paid a stipend. Subjective patient reporting of chronic pain was assessed initially in the study by the independent surgeon investigators. Any pain that did not prevent the patient from working was labeled mild to moderate.

### Phase 2 Questionnaire based follow-up obtained by data acquisition motorcycle driver at Mao & Moncion (MM)

At an investigator’s meeting it was decided that an inguinal herniorrhaphy validated follow-up questionnaire, developed in Scandinavia by Staerkle et al. [9] would render more data. However, the consensus of the four native Spanish speaking DR surgeon-investigators was that the questions were not culturally appropriate and required contextual modification with Spanish translation (Table 1). It was also decided that an employed motorcycle driver could better find patients for the MM group. The motorcycle driver obtained an informed consent and then connected the patient with the physician-investigator to answer the questionnaire. The employed motorcycle driver went to MM and 15 surrounding towns/villages in order to contact as many patients as possible. Fifteen trips were made by the driver and the approximate time spent with each trip was 10 hours with over 150 hours dedicated to identifying MM patients. The motorcycle driver, the MD patient data coordinator, and the patients were paid a stipend for their participation. 39 additional patients were found by the motorcycle driver and questioned by the data coordinator. The questionnaire modification was not anticipated in the original study protocol and because the English questionnaire version was well validated, a separate pilot validation study for the DR Spanish version was not thought feasible. Chronic pain was assessed subjectively by questionnaire question 3 (see Table 1).

**Table 1:** Cultural adjustment of 4 question questionnaire

**Scandinavian questionnaire**	
1. Is there a bulge? 2. Have you had another hernia operation? 3. Do you have chronic pain in the groin? 4. Does the pain prevent you from working?	
**Study Dominican Republic questionnaire English translation**	
1. After having surgery for your hernia, have you had any problems with your surgery? 2. Do you feel any discomfort in the surgery area? 3. What kind of annoyance? 4. Would you like a surgeon to see you?	
**Study Dominican Republic questionnaire Spanish translation**	
1. ¿Luego de haber sido operado de su hernia, ha tenido usted alguna situacion con su cirugia? 2. ¿Siente usted alguna molestia en el área de la cirugía? 3. ¿Que tipo de molestia? 4. ¿Le gustaría que un cirunajo lo vea?	

### Phase 1: In-person history and physical follow-up at ILAC

An in-country coordinator at ILAC contacted patients who had undergone a Lichtenstein inguinal hernia repair between 2014 and 2019. All 128 patients were called but 65% of phone numbers were incorrect. Voice mails were left when available, and WhatsApp was also utilized. If the patient did not answer initially the patient was called 5 more times and then put on the uncontacted list.

An independent DR board-certified surgeon obtained informed consent and then interviewed and examined 21 patients using the HRFU standardized questionnaire and examination form. Electronic spreadsheets were utilized for recording data. The surgeon-examiner and patients were paid a stipend for their participation.

### Phase 2 Questionnaire based follow-up obtained by data acquisition coordinatorat ILAC

ILAC health care promoters and the patient physician coordinator used the same questionnaire method as the MM group to identify 12 additional patients for the study. After obtaining informed consent, they connected the patient with the patient physician coordinator to answer the questionnaire. The health care promoters were more limited geographically thus patient recruitment was limited.

### Statistical analysis

Data was securely stored on cloud-based spreadsheets and was shared for data entry with the site physician coordinators. After exhausting all methods to identify additional patients, the study was closed on November 3, 2023. Data was then tabulated and triple checked. All data are presented for the overall patient cohort as well as stratified by phase and site. Continuous variables are reported as mean and standard deviation or median and interquartile range (IQR), compared using independent-samples t-test or Mann-Whitney test, respectively. Categorical data are presented as count and percent, compared using the chi-square test or Fisher’s exact test. Time-to-event outcomes were compared using the Kaplan-Meier method and log-rank test. All analyses were conducted using SAS v. 9.4 with two-tailed *p* <.05 used to indicate statistical significance.

## Results

### Moncion/Mao (MM)

Follow-up data was obtained from 14 patients using the in-person history and physical examination conducted by an independent surgeon and from 39 patients utilizing the 4-question questionnaire. A total of 53 of 160 patients from the MM site were included (33%). The average length of post-operative follow-up was 60 months. One hernia recurrence was identified through the questionnaire and no recurrences were identified in patients examined by the independent surgeon physical examination (1.9%). Four patients (7.5%) had chronic groin pain, 3 identified by the phone questionnaire and one by the physician examiner. None of the pain was considered severe and no patient experienced disability. There were no other complications.

### ILAC

Follow-up data was obtained from 21 patients using the in-person history and physical examination conducted by an independent surgeon and from 12 patients utilizing the 4-question questionnaire. The total follow-up from the ILAC site included 33 of 128 (26%) patients. The average length of follow-up was 52 months. One hernia recurrence was found on questionnaire and two by the independent surgeon physical examination (9.1%). Six patients (18.2%) had chronic groin pain, 4 identified by the phone questionnaire and two by the physician examiner. None of these were considered severe and no patient experienced disability. One of the chronic pain patients had a mesh granuloma, found by the examiner that required re-operation and one patient had a reoperation for a recurrent hernia.

### Overall

The average follow-up for all study patients was 56 months. Demographic statistical analysis between phase 1 and phase 2 of the study is shown in Tables 2, 3 and 4. No statistical tests of the Kaplan-Meier curves between phases could be conducted for recurrence due to there not being enough recurrences. However, when comparing outcomes between MM and ILAC, no statistically significant differences were indicated for overall recurrence rates (1.9% vs. 9.1%, *p* =.155), time-to-recurrence (log-rank *p* =.102), or overall post-operative pain (7.7% vs. 18.2%, *p* =.173).

**Table 2:** Overall statistics

Variable	Overall	
	*N*	Statistic
Age	86	57.1 ± 18.8
Biological Sex		
Female	53	8 (15.1)
Male		45 (84.9)
Satisfaction	35	10 [10–10]
Reoperations	86	2 (2.7)
Recurrences	86	4 (4.7)
Post-op Pain	86	10 (11.6)
Follow-up (months)	86	51 [48–66]
IQR		
Follow-up (months) Average	86	56

**Table 3:** Moncion & Mao Statistics

Variable	Overall		Phase 1		Phase 2		*p*
	*N*	Statistic	*N*	Statistic	*N*	Statistic	
Age	53	58.2 ± 19.7	14	52.2 ± 21.1	39	60.3 ± 19.0	0.221
Biological Sex							
Female	53	8 (15.1)	14	4 (28.6)	39	4 (10.3)	0.186
Male		45 (84.9)		10 (71.4)		35 (89.7)	
Satisfaction	14	10 [10–10]	14	10 [10–10]	0	-	-
Reoperations	53	0 (0.0)	14	0 (0.0)	39	0 (0.0)	-
Recurrences	53	1 (1.9)	14	0 (0.0)	39	1 (2.6)	1.000
Post-op Pain	53	4 (7.5)	14	1 (7.1)	39	3 (7.7)	1.000
Follow-up (months) IQR	53	56 [48–72]	14	46 [39–64]	39	53 [48–76]	0.592
Follow-up (months) Average	53	60					

**Table 4:** ILAC statistics

Variable	Overall		Phase 1		Phase 2		*p*
	*N*	Statistic	*N*	Statistic	*N*	Statistic	
Age	33	55.4 ± 17.3	21	60.8 ± 17.2	12	45.9 ± 13.3	0.010
Biological Sex							
Female	0	-	0	-	0	-	-
Male		-		-		-	
Satisfaction	21	10 [10–10]	21	10 [10–10]	0	-	-
Reoperations	21	2 (9.5)	21	2 (9.5)	0	-	-
Recurrences	33	3 (9.1)	21	2 (9.5)	12	1 (8.3)	1.000
Post-op Pain	33	6 (18.2)	21	2 (9.5)	12	4 (33.3)	0.159
Follow-up (months) IQR	33	48 [48–56]	21	48 [48–48]	12	52 [48–56]	0.395
Follow-up (months) Average	33	52					

A May 2024 literature search on low and middle income country long-term open inguinal hernia repair and the OPRS (in any setting) follow-up was performed using Medline and Embase. Only studies reporting specific quantitative follow-up times were included. Eleven studies were found reporting quantitative follow-up data, with an average of 11.1 months follow-up and a maximum of 36 months [10]. The same search engines were used to determine if the OPRS had been objectively evaluated by post training operative outcomes. One paper [11] followed up post-training patients for 6 months but none were found that evaluated operative results from trained surgeons for the operation learned.

## Discussion

Long-term surgical follow up has always posed a challenge even in the most ideal of settings. Nationwide registries enacted in single-payor and nationalized systems typically in resource rich countries have been the most successful at capturing a reflective picture of the efficacy of surgical intervention and the delivery of care. While no system is perfect, outcomes-based data and research is crucial to improving the quality of care. Arguably, this is of even greater importance in LMICs where patients are often disenfranchised of care, let alone high-quality surgical care. This study demonstrates that long term inguinal hernioplasty follow-up is possible, and long term health care data can be obtained in an LMIC setting - albeit with significant challenges and limitations.

Long-term follow-up is rare in LMIC settings for a multitude of reasons; patient transportation costs, difficult access to health care and surgeons, lack of patient education, contact information being quite transient, poor telecommunication or internet connectivity, unreliable electricity grids, limited resources for data acquisition and processing, and a lack of emphasis on the importance of data and outcomes as part of the delivery of healthcare. However, long term follow-up for inguinal hernia repair is the standard in the literature and carries even greater importance with mesh repairs because of the potential for delayed complications such as mesh infection, mesh erosion into adjacent organs, and disabling chronic inguinal pain. In a recent article published by Petersen et al. [9] time lapses between mesh implantation and excision were analyzed in 460 mesh explantations for complications. The 50th and 95th percentile for groin hernias was between 4.0 and 16.1 years substantiating the need for long term follow-up for hernia repair efficacy. Long term surgical follow-up may be enhanced by initiating a prospective study rather than a retrospective study so all of the following can be done proactively; (1) emphasize to the study medical staff the importance of long term hernia surgery follow-up, (2) effective and staged financial compensation for a motorcycle driver, (3) effective financial compensation for study patients to keep the doctor’s office appraised of changed phone numbers and mailing addresses, and (4) local study publicity to give the patients a sense of ownership and pride in participation.

One of the primary educational and programmatic focuses of HRFU is to build capacity, expertise, and strengthen systems in partner LMIC sites. While the expansion of surgical expertise, resources, and operative capacity is the primary objective to increase improved care to the poor, the importance of tracking outcomes, conducting research, presenting data in academic forums, and producing scientific publications is an essential endeavor to improve the local delivery of care and the local healthcare systems. It is understood that this enterprise is difficult, challenging, limited, and frustrating. The combination of persistence, local expertise, cultural acumen, and dedication are essential.

It became abundantly clear that the clinic visit approach would not render enough patients at both sites after months of effort. The modification to tracking outcomes using a follow-up questionnaire was implemented after the group of local investigators comprised of two Dominican surgeon partners, 3 Spanish speaking Dominican surgeon consultants, and a co-author advised a change. The local Dominican investigator team was instrumental in modifying the validated 4 question inguinal core outcome measure index (COMI) questionnaire for cultural and linguistic context (Table 1). The first question “Is there a bulge?” was changed because there is no good Spanish word for bulge and “the patient would require a lengthy explanation”. The second question “Have you had another hernia operation?” was changed because Dominican patients in the target demographic of our study are often not well-informed before or after operations and are not always aware of the operation previously performed. The fourth question, “Does the pain prevent you from working”?, was changed “because Dominicans often confuse pain with discomfort”. The culturally-modified inguinal COMI questionnaire is listed in Table 1.

**Fig. 1:**
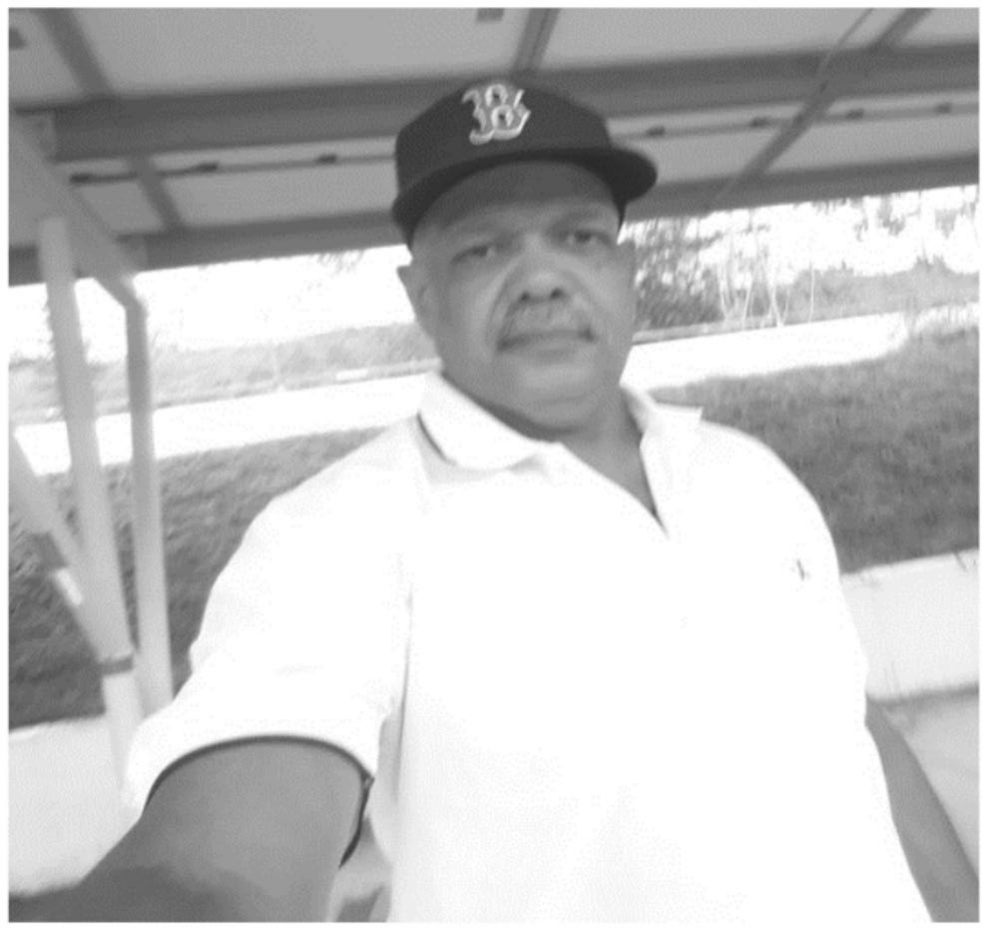
The motorcycle driver Señor Luis Rafael Morel

In this study, the importance of local teams, local resources, partnerships, and cultural acumen is clearly apparent as traditional means of follow-up were nearly futile. A limited number of patients would come in for a history and physical examination if given a modest financial incentive but only if this was convenient. Contacting patients by phone was challenging as phone numbers were often changed and voice mail was rarely available. A tenacious motorcycle driver (Fig. 1) with a good personal reputation in the community and exceptional perseverance was the most effective means of data acquisition, finding the majority of patients for the MM site. However, the number of located patients was low. Another probable method to enhance motorcycle driver follow-up is to pay the driver more and in stages. A separate stipend for all of the following; (1) locating the patient, (2) obtaining the written consent, (3) taking a video of the signing of the consent, (4) completing the patient phone call with the data coordinator and (5) returning the signed consent to the data coordinator.

Without local partnerships and resources along with exemplary individual effort, research in these settings would be impossible. While the following anecdotes are not necessarily scientific, they illustrate that all research succeeds through diligence, persistence, and sometimes creative problem solving. The following are quotes from the data-acquisition motorcycle driver reflecting his tenacity and diligence. “The first step of my day was to commend myself to God. As soon as I got to the assigned city, I would find neighborhood leaders, the city’s mayor, and church pastors. If the patient was of a younger age, I would approach high school teachers, college principals, and find their student records. I was always grateful the people always trusted me and collaborated with me.

**Fig. 2:**
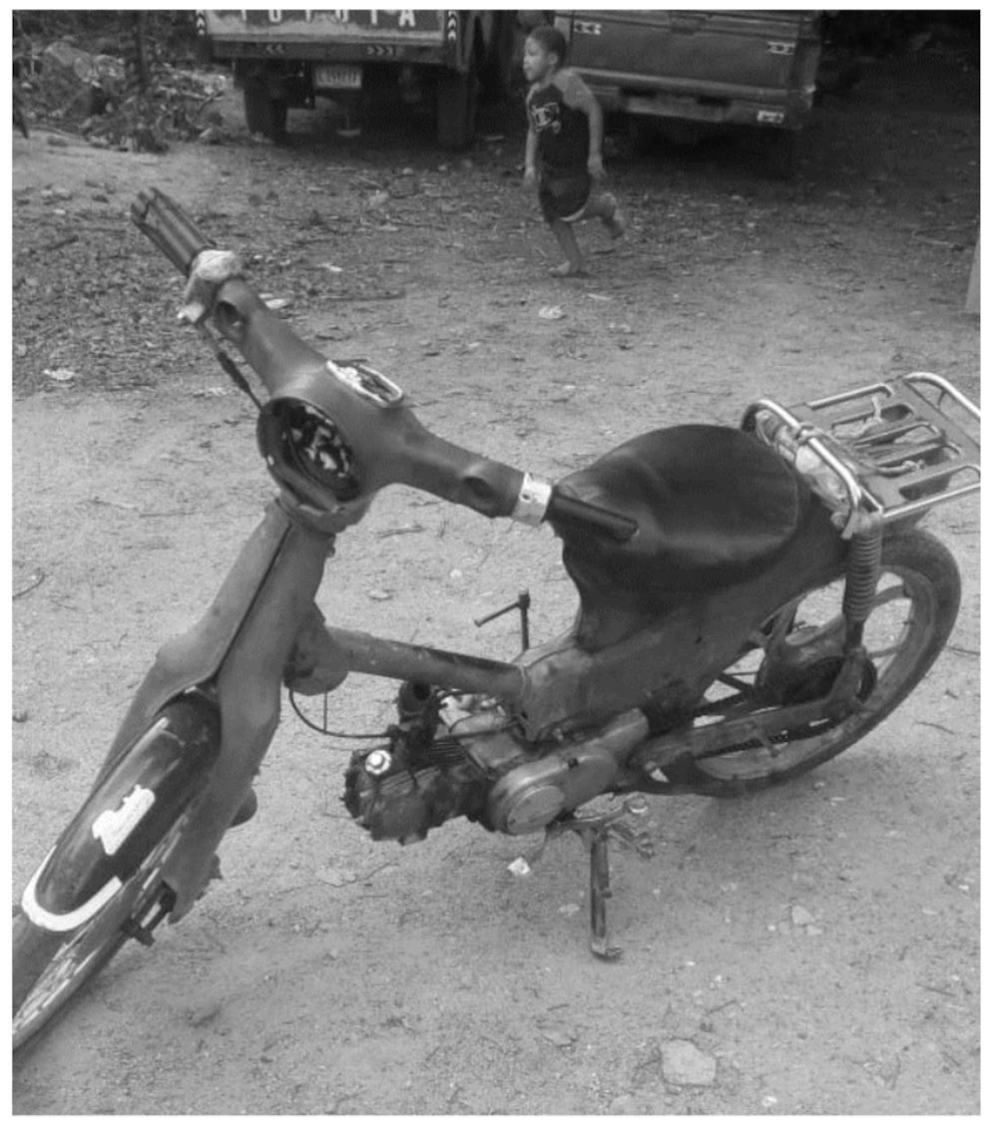
The motorcycle used. Note no headlight

I would ride home very late because the weather slowed my drive. My motorcycle doesn’t have any front light (Fig. 2) so every patient had to be located during daylight. I tried to visit every address on the list that was provided to me”.

When the driver was asked what the most rewarding part of the job was, he replied, “When patients realized that someone from the medical field wanted to follow up, this put a smile on their faces knowing that the doctor cared about their surgery so many years after.” The 65 year old driver was determined to “help his brother”, one of the Dominican surgeons.

Through this exhaustive, Herculean effort, we are able to report the longest interval follow-up of outcomes after inguinal hernia repair in a LMIC setting. We also demonstrate for the first time, post-operative patient outcomes from trainees that have been assessed for competency using the OPRS as part of their training program. The proficiency and competency demonstrated by our two local Dominican surgeons in modern Lichtenstein mesh-based inguinal hernia repair, translated to good long-term outcomes 5 years later in a resource-disadvantaged patient population.

In addition to strengthening clinical care, healthcare delivery, and research activities, several other initiatives have been adopted to improve the local delivery of surgical care. Perioperative and postoperative follow-up protocols have been implemented at both the ILAC center and MM practices. Specific discharge instructions and handouts are given to each patient delineating post-operative care, potential complications, and direct physician contact information in the event of issues. A follow-up examination is arranged at 7–10 days post-operation with a DR health care provider. For any post-operative concerns with ILAC patients operated by the international team, a text or email with clinical photographs were sent to the international team leader and if concerns remained, an appointment for a local DR surgeon partner was arranged. This continuity emphasizes the crucial role of local surgical partners to ensure clinical care and strengthen the local surgical infrastructure.

Another systematic benefit of the HRFU training program has been the adoption of routine local anesthesia for open inguinal hernia repair. Local anesthesia administration was taught during the HRFU training program at MM. The two DR surgeons now administer it almost routinely in their daily hernia practice.

In addition, the MM site has initiated a local registry of operated patients. These records were converted to record books that included a patient identification number, social security number, the patients’ sex, age, ethnicity, the operative anesthesia method, the operation performed, and the acting surgeon’s name. A team of data technicians and computer scientists was employed to develop a cloud-based database from the record books, to be used for patient follow-up and research purposes.

The study limitations include the small patient sample size which limits the results validity, the modification of the validated 4 question COMI herniorrhaphy questionnaire for cultural and linguistic context, only two trained surgeons were included in this study cohort, the unusual passion the motorcycle driver exhibited with his duties may represent unrealistic expectations for utilizing a motorcycle driver, and different independent surgeon examiners were used in phase 1.

The outcome results of the ILAC team are below the anticipated standard of care. The concordance between patient reported outcome and a clinical examination proven recurrence is variable. The international hernia experts performed a Lichtenstein repair as stated in the operative note on each patient included in this study, but there were technical variations that were performed in these non-teaching cases that may deviate from the optimal technique described by Dr. Parviz Amid. The available spreadsheet records recorded the answers required of the surgeon in an abbreviated ILAC operative note. Intraoperative difficulty was not recorded. The postoperative care unit (PACU) record does record additional complications in the PACU such as wound hematoma or urinary retention but records for further short term follow-up were not obtained. None of the ILAC recurrent patients had recorded intraoperative complications, but one of the Lichtenstein operation recurrences had an operative time of only 18 min and it was learned later only absorbable suture was used for mesh fixation in another recurrent hernia case. At MM the same is true for operative notes. These patients were routinely seen 10 days postoperatively by the operating surgeon but none of these records were reviewed. Otherwise they were not followed until the study was initiated.

The formally trained local surgeons explicitly performed an Amid-modified Lichtenstein repair utilizing all of the individual steps as taught by our HRFU trainers in our formal metrics-based training program. The strict adherence to each of these technical steps likely accounts for some of these differences in outcome.

The HRFU surgeon coordinator has been consistently conscientious about pre-operative evaluation and peri-operative care and follow-up of patients served by the organization. The organization has explicitly taken clinical, logistic and financial responsibility for any complications with each of these patients. From the inception of this program, there have been only 4 of 1,800 hernia operations at ILAC (2004 to 2024 except 2021 due to COVID) that have required postoperative hospitalization (aspiration pneumonia, a post-operative inguinal hematoma, hypoglycemia and urinary retention) and no deaths. The HRFU surgeon coordinator recalls 4 patients returning with a recurrent hernia during his 20 years of work in the Dominican Republic but other recurrences have most likely occurred. The surgeon coordinator as team leader, admits he did not spend enough time confirming that each international expert complied with all the surgical technical details recommended by the Lichtenstein Clinic. Conversely, the more favorable results from the local surgeons is a source of pride and reflection of the effort to build local capacity and expertise.

## Conclusion

The study reports the longest duration of follow-up after inguinal hernia repair performed in a LMIC setting. We also report post-operative patient outcomes from trainees that have been assessed for competency using the OPRS, demonstrating for the first time a linkage between assessed validated proficiency to clinical patient outcomes. The proficiency and competency demonstrated by our two local Dominican surgeons in modern Lichtenstein mesh-based inguinal hernia repair translated to good long-term outcomes 5 years later in a resource-disadvantaged patient population. The HRFU training method is likely satisfactory, although the sample size is small. The hernia recurrence rates and incidence of chronic pain rate were acceptable at the training site. This study enabled patients with inguinodynia or recurrence to be followed up and instilled in patients a confidence that there are efforts to ensure that the care they received is of high-quality with a desire and mechanism to continually improve. The infrastructure and expertise developed for this study has enabled the initiation of a larger prospective study in the same communities with patient education at the time of surgical intervention and systematic intermittent follow-up. Obtaining long-term follow up in LMIC settings is a challenge requiring creativity and local resourcefulness. It is an essential endeavor to ensure surgical quality and improve the system wide standard of care worldwide.

## Data Availability

personal communication and excel spreadsheet on google documents.
